# Book Notices

**Published:** 1883-01-20

**Authors:** 


### *BOOK NOTICES.*

THE PHARMACOPŒA OF THE UNITED STATES OF AMERICA—Sixth Decennial Revision, by Authority of the National Convention for Revising the Pharmacopœa, held at Washington, A. D. 1880. New York: Wm. Wood & Co. McGarity & Laird, Agents, Atlanta, Ga.

This long expected work is now before the profession. Though designed specially for the Pharmacist, it is yet highly interesting and useful to the medical man. Certain new and progressive features will be observed in the work. The abstracts constitute a new and separate class of preparations. They may be termed "powdered extracts." Elixers are not recognized. The alkaloids are made to terminate in *ina*, as quina, morphina, etc. Temperature is expressed both in Fahrenheit and contegrude scale. Both the mitric and common measures are used. We regret to notice that the doses are not given.

The work contains 488 octavo pages, and is sold in muslin at $4.00; in leather at $5.00

